# Oxidative Stress in Glaucoma: From Pathogenic Mechanisms to Emerging Antioxidant Therapies

**DOI:** 10.3390/antiox15060751

**Published:** 2026-06-14

**Authors:** Akiko Hanyuda, Satoru Tsuda, Naoki Takahashi, Masataka Sato, Kota Sato, Noriko Himori, Toru Nakazawa

**Affiliations:** 1 Department of Ophthalmology, Keio University School of Medicine, Shinjuku 160-8582, Japan; akihanyu@keio.jp; 2 Department of Ophthalmology, Tohoku University Graduate School of Medicine, Sendai 980-8575, Japan; 3 Division of Ophthalmic Precision Medicine Development, United Centers for Advanced Research and Translational Medicine (ART), Tohoku University Graduate School of Medicine, Sendai 980-8575, Japan; 4 Department of Aging Vision Healthcare, Tohoku University Graduate School of Biomedical Engineering, Sendai 980-8575, Japan; 5 Department of Retinal Disease Control, Tohoku University Graduate School of Medicine, Sendai 980-8575, Japan; 6 Department of Advanced Ophthalmic Medicine, Tohoku University Graduate School of Medicine, Sendai 980-8575, Japan

**Keywords:** glaucoma, oxidative stress, reactive oxygen species, trabecular meshwork, retinal ganglion cells, Nrf2, nicotinamide, neuroprotection, antioxidants, mitochondrial dysfunction

## Abstract

Glaucoma is the leading cause of irreversible blindness worldwide and is characterized by progressive retinal ganglion cell (RGC) loss and optic nerve degeneration. While elevated intraocular pressure (IOP) remains the primary modifiable risk factor, a certain proportion of patients continue to deteriorate despite adequate IOP control, pointing to IOP-independent mechanisms of neurodegeneration. Oxidative stress—defined as an imbalance between the production of reactive oxygen species and the capacity of endogenous antioxidant defenses—has emerged as a central, multi-tiered contributor to glaucoma pathogenesis. In the anterior segment, chronic oxidative damage to the trabecular meshwork impairs aqueous humor outflow and drives IOP elevation. In addition, oxidative stress may impair ocular biomechanical integrity, including corneal hysteresis and lamina cribrosa, resulting in heightened susceptibility to IOP fluctuations. In the posterior segment, oxidative stress directly contributes to mitochondrial damage and vascular endothelial injury, leading to RGC apoptosis. The nuclear factor erythroid 2-related factor 2 (Nrf2)/Kelch-like ECH-associated protein 1 (Keap1) pathway coordinates the principal endogenous antioxidant response, while nicotinamide adenine dinucleotide (NAD^+^) depletion links redox imbalance to metabolic vulnerability of RGCs. This narrative review synthesizes evidence published up to March 2026 on the molecular mechanisms of oxidative stress in glaucoma, the role of biomarkers in aqueous humor and systemic circulation, and the translational landscape of antioxidant-based neuroprotection—including nicotinamide, coenzyme Q10, alpha-lipoic acid, and Nrf2-activating compounds. We highlight gaps between preclinical promise and clinical evidence, and outline priorities for future randomized controlled trials.

## 1. Introduction

Glaucoma constitutes a heterogeneous group of progressive optic neuropathies and represents the leading cause of irreversible blindness globally. Epidemiological projections estimate that approximately 111.8 million individuals aged 40–80 years will be affected by 2040, imposing an enormous burden on healthcare systems [1]. The cardinal feature of glaucoma is the apoptotic loss of retinal ganglion cells (RGCs) and their axons, resulting in characteristic structural changes at the optic nerve head and progressive visual field loss [2].

Elevated intraocular pressure (IOP) remains the only proven modifiable risk factor and the principal target of all current pharmacological and surgical interventions. However, a sizeable subset of patients—including a fraction of primary open-angle glaucoma (POAG) patients, particularly those with normal-tension glaucoma (NTG)—continue to lose vision despite IOP within the normal range or following its successful reduction [3]. This observation has catalyzed intense investigation into IOP-independent pathogenic pathways, among which oxidative stress has attracted particular attention.

Reactive oxygen species (ROS) are inevitable by-products of aerobic metabolism, generated predominantly by the mitochondrial electron transport chain (ETC), NADPH oxidase (NOX) family enzymes, xanthine oxidase, and other sources [4]. Under physiological conditions, cellular antioxidant systems—including superoxide dismutase (SOD), catalase, glutathione peroxidase (GPx), and the transcription factor Nrf2—maintain redox homeostasis. Oxidative stress arises when this balance is disturbed, leading to cumulative damage to lipids, proteins, and nucleic acids [5].

**Figure 1:**
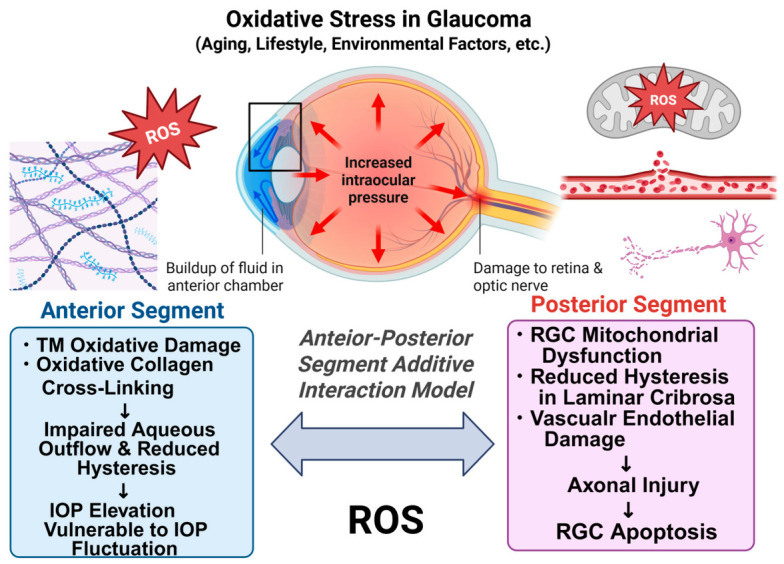
Dual-compartment model of oxidative stress in glaucoma. The anterior–posterior segment additive interaction model posits that oxidative stress–induced biomechanical stiffening in the anterior eye amplifies IOP-related stress transmission, while concurrent neurovascular vulnerability in the posterior eye lowers the threshold for damage, together resulting in cumulative RGC degeneration (created in BioRender. Hanyuda, A. (2026) https://BioRender.com/9wwipr5 (accessed on 3 June 2026)). IOP: intraocular pressure; RGC, retinal ganglion cells; ROS: reactive oxygen species; TM: trabecular meshwork.

The eye is particularly vulnerable to oxidative insult. Its unique microenvironment combines continuous exposure to visible and ultraviolet radiation, high oxygen tension in the anterior chamber, a demanding metabolic profile in RGCs, and a relatively limited regenerative capacity [6]. Evidence from human ocular samples and animal models consistently demonstrates ROS accumulation and oxidative biomarker elevation across all anatomical compartments relevant to glaucoma: the trabecular meshwork (TM), the optic nerve head (ONH), and the RGC layer [7,8]. In the anterior segment, oxidative stress may impair ocular biomechanical integrity, including corneal hysteresis, due to oxidative collagen cross-linking, leading to increased vulnerability to IOP fluctuation [7,8]. In the posterior segment, ROS may directly induce cellular mitochondrial damage and vascular endothelial dysfunction [7,8]. These additive interactions between anterior and posterior segments via ROS may collectively induce glaucomatous neurodegeneration (Figure 1).

This narrative review was conducted according to the following search and selection strategy. PubMed/MEDLINE, Scopus, and Web of Science were searched using the following query as the core string: (glaucoma OR primary open-angle glaucoma OR normal-tension glaucoma) AND (oxidative stress OR reactive oxygen species OR ROS OR lipid peroxidation OR mitochondrial dysfunction OR Nrf2 OR antioxidant). Subsidiary searches incorporated topic-specific terms including TM, RGC, aqueous humor biomarker, nicotinamide, coenzyme Q10, sulforaphane, MitoQ, and NAD^+^. We restricted the inclusion to papers written in English; the search was limited to articles published up to March 2026, with seminal earlier works retained where necessary to contextualize foundational concepts. Titles and abstracts were screened independently by the authors, and full texts were retrieved for any study reporting original data on oxidative mechanisms, redox biomarkers, or antioxidant interventions in human glaucoma tissue, animal models of glaucoma, or clinical trials. Review articles, meta-analyses, and position statements were included when they synthesized primary evidence directly relevant to the four domains addressed here: (i) molecular and cellular mechanisms by which oxidative stress contributes to TM dysfunction and IOP elevation; (ii) pathways linking ROS to RGC death and optic nerve degeneration; (iii) biomarkers of oxidative stress measurable in aqueous humor, vitreous, and systemic compartments; and (iv) the current and emerging landscape of antioxidant-based neuroprotective strategies. Studies were excluded if they addressed oxidative stress solely in the context of unrelated ocular conditions (e.g., age-related macular degeneration, diabetic retinopathy) without a specific glaucoma focus, or if outcome data were insufficient to draw mechanistic or clinical conclusions.

This narrative review aims to provide a comprehensive synthesis of the current evidence on: (i) the molecular and cellular mechanisms by which oxidative stress contributes to TM dysfunction and IOP elevation; (ii) the pathways linking ROS to RGC death and optic nerve degeneration; (iii) biomarkers of oxidative stress measurable in aqueous humor, vitreous, and systemic compartments; and (iv) the current and emerging landscape of antioxidant-based neuroprotective strategies.

## 2. Sources and Chemistry of Reactive Oxygen Species in the Glaucomatous Eye

Free radicals are chemical entities characterized by one or more unpaired electrons and are produced constitutively at rates estimated to approach 10^11^ molecules per day in normal human cells [9]. Oxygen-centered radicals—collectively termed ROS—account for approximately 95% of the free radical burden and include superoxide anion (O_2_^•^^−^), hydrogen peroxide (H_2_O_2_), hydroxyl radical (^•^OH), peroxyl, alkoxyl, and singlet oxygen species [10]. Reactive nitrogen species, particularly peroxynitrite (ONOO^−^) formed by the reaction of O_2_^•−^ with nitric oxide (NO^•^), also participate in oxidative damage cascades relevant to glaucoma, although they are outside the primary scope of this review.

### 2.1. Mitochondrial Origins

The mitochondrial ETC, specifically complexes I (NADH: ubiquinone oxidoreductase) and III (ubiquinol–cytochrome c reductase), represents the dominant intracellular source of superoxide during normal respiration. In RGCs—which possess exceptionally high metabolic demands due to the long, unmyelinated axon segments traversing the lamina cribrosa—mitochondrial density is correspondingly elevated, making these neurons intrinsically susceptible to mitochondria-derived oxidative stress [11]. Experimental elevation of IOP in rodent models reliably produces an early, transient surge in retinal ROS detectable within several weeks, followed by sustained oxidative stress coinciding with the onset of RGC loss [12]. Mitochondrial complex I defects have been identified in the TM of patients with POAG, creating a vicious cycle in which impaired ETC efficiency amplifies superoxide production [13].

### 2.2. NADPH Oxidase and Non-Mitochondrial Sources

The NOX family of enzymes generates superoxide as its primary product upon stimulation by growth factors, cytokines, and mechanical stress. NOX2 and NOX4 isoforms have been detected in TM cells, RGCs, and retinal Müller glia, and their activity is upregulated under conditions of elevated IOP and oxidative priming [4]. The endoplasmic reticulum (ER) and peroxisomes also contribute to the cellular ROS burden, particularly under conditions of proteostatic stress—a feature increasingly recognized in glaucomatous TM cells, which exhibit accumulation of misfolded extracellular matrix (ECM) proteins and impaired autophagic flux [14].

### 2.3. Exogenous and Environmental Contributors

Beyond endogenous sources, the anterior segment of the eye is uniquely exposed to exogenous oxidants. Cumulative ultraviolet radiation exposure, photoexcitation of aqueous humor chromophores, cigarette smoke-derived oxidants, and systemic pro-oxidant states (e.g., diabetes mellitus, hypertension, and ageing) all augment ocular ROS levels [6]. Notably, physiological oxygen tension in the aqueous humor of the anterior chamber is significantly higher than in most tissues, creating a high-oxygen milieu that, while necessary for avascular anterior segment metabolism, amplifies the risk of oxidative TM injury [15].

## 3. Oxidative Stress and TM Dysfunction

The TM is a specialized, porous connective tissue located at the iridocorneal angle through which approximately 70–90% of aqueous humor drains into Schlemm’s canal and the episcleral venous circulation. Its resistance to outflow is the principal determinant of IOP, and dysfunction of TM cells (TMCs) is regarded as the proximate cause of IOP elevation in POAG [16]. A recent transcriptomic integration of glaucomatous TM dataset has nominated oxidative-stress signature genes converging on NF-κB/TNF inflammatory pathways, reinforcing the redox–inflammation interface in TM dysfunction [17]. Oxidative damage to the TM is now recognized as a critical early event in glaucoma pathogenesis.

### 3.1. Direct Oxidative Damage to TM Cells

Multiple lines of evidence link ROS to TM dysfunction. First, oxidative DNA damage, assessed by 8-hydroxy-2′-deoxyguanosine (8-OHdG) immunoreactivity, is significantly elevated in TM tissue from POAG donors relative to age-matched controls, and this elevation correlates with IOP magnitude and the severity of visual field loss [18]. Second, lipid peroxidation products—including malondialdehyde (MDA) and 4-hydroxynonenal (4-HNE)—are detectable in TM cells and aqueous humor of glaucoma patients, indicating membrane oxidation and impaired barrier function [19]. Third, oxidative carbonylation of TM proteins, including fibronectin, laminin, and collagen IV—essential ECM constituents of the trabecular beams—leads to their cross-linking, stiffening, and reduced degradability, increasing outflow resistance [20].

Patients with POAG demonstrate a total reactive antioxidant potential (TRAP) in aqueous humor that is lower than that of cataract controls—the comparator group used in the key study—suggesting a chronically compromised local antioxidant reserve [10]. Systemic oxidative stress markers show consistent elevation of MDA and reduction in GSH in POAG patients [21]. However, the direction of serum SOD, GPx, and catalase activities is inconsistent across studies—with several reporting compensatory upregulation rather than reduction—and the statement that these enzymes are ‘consistently reduced’ is not supported by the literature as a whole [21,22].

### 3.2. Proteasome Inhibition and Autophagic Dysfunction

Chronic ROS exposure impairs the ubiquitin–proteasome system in TMCs, reducing their capacity to degrade oxidatively modified proteins. This proteasome inhibition results in the accumulation of cross-linked protein aggregates within TM cells, further compromising cellular function and outflow facility [14]. Simultaneously, chronic oxidative stress activates the ER stress response via the unfolded protein response, upregulating pro-apoptotic mediators including ATF4 and CHOP/DDIT3 in TM cells and glaucomatous TM tissues [23]. These mechanisms contribute to TMC senescence and progressive cell loss [14]. Aging is itself a major amplifier of this vulnerability: age-related reductions in ocular blood flow at the optic nerve head and macula [24,25], progressive axonal attrition in the visual pathway [26,27], and accumulating mitochondrial DNA damage compound the susceptibility of RGCs to oxidative insult even before overt glaucomatous injury is detectable [28].

### 3.3. Senescence and Myocilin

TM cell senescence, driven in part by cumulative oxidative DNA damage and telomere erosion, is now considered a hallmark of POAG. Senescent TMCs exhibit a senescence-associated secretory phenotype (SASP), releasing pro-inflammatory cytokines (IL-6 and IL-8) that further oxidize and degrade the trabecular ECM [29]. Myocilin (MYOC), encoded by a gene mutated in approximately ~5% of POAG cases, has been shown to misfold under oxidative conditions, with misfolded myocilin accumulating in the endoplasmic reticulum, activating unfolded protein response, and promoting TMC apoptosis [30]. This molecular crosstalk between oxidative stress, proteostasis, and genetic glaucoma susceptibility underscores the integrative nature of TM pathology.

### 3.4. MicroRNA Regulation of Redox Balance in the TM

An emerging body of evidence implicates non-coding RNAs as important modulators of the oxidative stress response in TM cells. Several microRNAs (miRNAs), including miR-7, miR-24, miR-27a, miR-29b, and miR-4295, exert antioxidant effects in TM cells by suppressing pro-oxidant regulators or upregulating Nrf2-target genes [31]. Conversely, dysregulation of miRNA expression under conditions of chronic oxidative priming may render TM cells less capable of mounting an adequate antioxidant response. These findings open a potential avenue for miRNA-based therapeutics targeting the anterior segment, although clinical translation remains at an early stage.

## 4. Oxidative Stress and RGC Death

The posterior segment consequences of oxidative stress in glaucoma are arguably more clinically significant, as they operate independently of IOP and directly determine the trajectory of vision loss. RGC apoptosis in glaucoma is multi-factorial, involving mechanical compression of axons at the lamina cribrosa, disrupted retrograde transport of neurotrophic factors, excitotoxic glutamate accumulation, neuroinflammation, and ischemia—all of which converge on ROS generation as a proximate executioner of cell death [32].

### 4.1. Mitochondrial Dysfunction and the Bioenergetic Crisis

RGCs possess an exceptionally high mitochondrial density at the unmyelinated axon segments within the ONH, reflecting their intense ATP requirements. When IOP elevation compresses ONH microvasculature, the resulting ischemia–reperfusion cycle generates a burst of superoxide via xanthine oxidase and mitochondrial complex I reverse electron transport [11]. Subsequent mitochondrial membrane permeabilization activates the intrinsic apoptosis pathway via cytochrome c release and caspase-9 activation. Crucially, this cascade can be self-reinforcing: oxidatively damaged mitochondrial DNA (mtDNA) encodes dysfunctional ETC subunits, further amplifying ROS production in a feed-forward loop [28].

Nicotinamide adenine dinucleotide (NAD^+^) is a central cofactor for both oxidative phosphorylation and the sirtuin family of deacylases (SIRT1–SIRT7) that regulate mitochondrial biogenesis, autophagy, and DNA damage responses. An age-dependent decline in retinal NAD^+^ levels has been documented in both rodent models and glaucoma patients, making RGCs metabolically vulnerable to superimposed IOP stress [33]. NAD^+^ depletion is now viewed as a unifying metabolic vulnerability that connects the bioenergetic and redox dimensions of glaucomatous neurodegeneration.

### 4.2. Lipid Peroxidation and Membrane Damage

RGC membranes are enriched in polyunsaturated fatty acids—particularly docosahexaenoic acid (DHA)—which are highly susceptible to peroxidation by ^•^OH and lipid peroxyl radicals. Peroxidation generates 4-HNE and MDA as reactive aldehydes that covalently adduct mitochondrial proteins—including respiratory chain subunits and the adenine nucleotide translocase (ANT)—inhibiting ETC function and dissipating the inner mitochondrial membrane potential (ΔΨm), thereby sensitizing the mitochondrial permeability transition pore (mPTP) to Ca2+-induced opening; sustained mPTP opening in turn triggers cytochrome c release and irreversible commitment to the intrinsic apoptotic pathway [34]. Elevated levels of MDA have been detected in the aqueous humor and retina of glaucoma patients, and their concentrations correlate with the degree of optic nerve damage [19].

### 4.3. Excitotoxicity and Nitric Oxide

Glutamate excitotoxicity—driven by excessive activation of N-methyl-D-aspartate (NMDA) receptors on RGCs—contributes to calcium influx and the activation of neuronal nitric oxide synthase. The resulting overproduction of NO^•^ reacts with O_2_^•−^ to form the potent oxidant peroxynitrite, which nitrates proteins, oxidizes lipids, and induces DNA strand breaks [35]. This pathway is particularly relevant in NTG, where ischemia-mediated excitotoxicity may be the dominant driver of RGC loss in the absence of significant IOP elevation. A murine NMDA-induced retinal injury model demonstrated that a plant-derived antioxidant supplement attenuated RGC loss, providing proof-of-concept evidence that scavenging excitotoxicity-driven ROS is sufficient to confer structural neuroprotection [36].

### 4.4. Neuroinflammation and the Redox–Immune Interface

Glaucomatous neurodegeneration is increasingly understood as an inflammatory process, with microglial activation and complement deposition at the ONH preceding detectable RGC loss in animal models [37]. ROS activate the NLRP3 inflammasome and NF-κB pathway in retinal microglia and astrocytes, driving the release of TNF-α, IL-1β, and IL-6 that create a cytotoxic environment for RGCs. Conversely, neuroinflammatory cytokines upregulate NOX2 in glial cells, sustaining ROS production in a self-amplifying inflammatory–redox cycle [38]. Resveratrol has recently been shown to attenuate retinal ischemia–reperfusion injury in rodents by simultaneously suppressing the NLRP3 inflammasome and activating the Keap1/Nrf2/HO-1 pathway [39], highlighting the mechanistic overlap between antioxidant and anti-inflammatory therapeutic strategies. The para-inflammatory response, which normally serves a homeostatic function, thus becomes maladaptive under conditions of chronic oxidative priming, accelerating rather than restraining RGC degeneration.

## 5. Endogenous Antioxidant Defenses: The Nrf2/Keap1/ARE Pathway

The Nrf2/Keap1/antioxidant response element (ARE) axis constitutes the master regulatory system for inducible antioxidant gene expression in mammalian cells. Understanding the activation and attenuation of this pathway in glaucomatous tissues is fundamental to both the interpretation of pathogenic evidence and the design of therapeutic interventions.

### 5.1. Molecular Mechanism of Nrf2 Activation

The molecular foundations of the Nrf2/ARE pathway were established by Itoh and colleagues, who first characterized the Nrf2/small Maf heterodimer as the transcriptional activator binding ARE sequences to induce phase II detoxifying enzyme genes [40], and subsequently demonstrated that Keap1 represses Nrf2 nuclear activation by direct binding to its amino-terminal Neh2 domain [41]. Under basal conditions, Nrf2 is retained in the cytoplasm by its repressor Keap1, which bridges Nrf2 to the Cullin 3 (Cul3)-based E3 ubiquitin ligase complex, targeting it for continuous proteasomal degradation. Reactive cysteines on Keap1 (C151, C273, C288) act as electrophile sensors; their oxidation or adduction by ROS or electrophilic activators induces a conformational change that prevents Nrf2 ubiquitination, allowing it to accumulate, translocate to the nucleus, and bind to ARE sequences [42]. Downstream transcriptional targets include NADPH quinone oxidoreductase 1 (NQO1), heme oxygenase-1 (HO-1), SOD2, GPx, thioredoxin reductase (TrxR), glutathione S-transferase (GST), and ferritin heavy chain—collectively constituting a broad-spectrum antioxidant arsenal. Flavonoids, including quercetin, protect RGCs from oxidative stress-induced death in vitro through this pathway [43], and quercetin has further been shown to upregulate the mitochondrial antioxidant enzymes peroxiredoxin 3 and 5 via the Nrf2/NRF1 axis in ocular cells [44].

Nrf2 activation in the context of elevated IOP has been demonstrated in the murine microbead occlusion model: retinal ROS peak at one to two weeks post-IOP elevation and trigger phosphorylation of Nrf2 via the PI3K/AKT pathway, with concomitant upregulation of antioxidant gene transcription and protein levels of PRDX6, SOD3, and GPx1 [12]. Critically, mice lacking Nrf2 showed greater ROS accumulation, diminished antioxidant gene induction, and earlier RGC loss—establishing that the Nrf2/ARE response is not merely a correlate but a genuinely neuroprotective mechanism. Building on this mechanistic foundation, Fujita et al. demonstrated that adeno-associated virus (AAV)-mediated overexpression of NRF2 under the stress-responsive Mcp-1 promoter—which is selectively activated in injured RGCs preceding cell death—conferred RGC protection equivalent to constitutive cytomegalovirus (CMV) promoter-driven NRF2 expression in a murine optic nerve crush model, while substantially reducing off-target cellular stress responses and death of healthy, uninjured RGCs observed with the non-selective CMV promoter [45]. This targeted gene therapy strategy illustrates how spatially and temporally regulated Nrf2 activation may be harnessed for neuroprotection while minimizing adverse effects on healthy retinal neurons—a consideration of direct relevance to future clinical translation.

### 5.2. Nrf2 in TM Cells and Age-Related Decline

In TM cells, Nrf2 expression is downregulated relative to normal controls, with Nrf2 overexpression restoring viability and reducing apoptosis via regulation of BCL-2 and Bax [46]. More broadly, the adaptive Nrf2/ARE antioxidant response to oxidative challenge declines with advancing age across multiple cell types [47], a principle that likely contributes to the age-dependence of POAG risk and the heightened susceptibility of aged TM cells to H_2_O_2_-induced oxidative injury.

### 5.3. Cell-Type Specificity of the Nrf2 Response

A recent study using cell-type-specific conditional knockout mice (Nrf2^fl/fl^ crossed with AAV-Cre constructs targeting either RGCs or glial cells) demonstrated that the primary endogenous antioxidant response in the retina under ocular hypertension is predominantly driven by glial cells rather than RGCs themselves [48]. Selective deletion of Nrf2 from Müller glia and astrocytes exacerbated RGC loss and optic nerve degeneration, while RGC-specific deletion had a comparatively modest effect. These findings suggest that therapeutic strategies should consider glial Nrf2 as a preferred target, and that neuroprotection may be achievable by reinforcing the supportive antioxidant capacity of the glial environment rather than—or in addition to—directly targeting RGCs.

## 6. Biomarkers of Oxidative Stress in Glaucoma

The availability of clinically accessible biomarkers that reflect the degree of oxidative burden in glaucomatous eyes would enable patient stratification, disease monitoring, and the evaluation of antioxidant therapies in clinical trials. Multiple compartments—aqueous humor, vitreous, serum, and peripheral blood cells—have been investigated. Table 1 summarizes the oxidative stress biomarkers in glaucoma.

### 6.1. Aqueous Humor Biomarkers

Aqueous humor provides a window into the biochemical microenvironment of the anterior segment and, to a lesser extent, the posterior segment. Ferreira and colleagues demonstrated that aqueous humor TRAP is significantly reduced in POAG patients relative to cataract controls, while SOD and GPx activities are paradoxically elevated—a pattern interpreted as a compensatory but ultimately insufficient response to chronic oxidative stress [10]. Nucci and colleagues subsequently confirmed that aqueous humor MDA concentrations are significantly elevated alongside reduced total antioxidant capacity (TAC), with parallel changes observed in blood samples from the same patients, suggesting that the redox imbalance extends beyond the ocular compartment [19]. More recently, Wu and colleagues quantified 8-OHdG in AH from 173 surgical patients and found significantly higher levels in POAG than in cataract controls; notably, Black patients with severe-stage disease exhibited greater 8-OHdG elevation than White patients, a disparity that correlated with enhanced reactive oxygen species production in cultured TM cells from healthy Black donors [49].

### 6.2. Serum/Plasma Biomarkers

Evidence from multiple independent cohorts demonstrates that glaucoma is accompanied by measurable systemic oxidative stress. Rokicki and colleagues found significantly elevated serum MDA and reduced total SOD activity in POAG patients compared with non-glaucomatous cataract controls, establishing a systemic pro-oxidant shift independent of cataract [21]. Gherghel and colleagues showed that blood GSH was significantly lower in both POAG and NTG patients than in age-matched controls, with the degree of depletion comparable across subtypes, suggesting a shared antioxidant deficit irrespective of IOP status [22]. In a Japanese NTG cohort, Yuki and colleagues reported selectively reduced serum vitamin C alongside paradoxically elevated uric acid, with other antioxidant vitamins unaffected—a pattern consistent with IOP-independent oxidative dysregulation specific to NTG [50].

Plasma nicotinamide is reduced by approximately 30% in POAG patients relative to controls—a finding replicated in an independent cohort—implicating upstream NAD^+^ depletion and mitochondrial dysfunction as systemic contributors to RGC susceptibility [51]. Serum biological antioxidant potential (BAP), reflecting integrated ferric-reducing antioxidant capacity, is consistently lower in POAG and exfoliation syndrome patients than in controls [52], and its association with estimated RGC counts and optic nerve head blood flow is modulated by both age and sex, being most pronounced in male patients aged 65 years or younger [53,54]. Oxidative DNA damage markers (urinary 8-OHdG) and skin autofluorescence are independently associated with reduced ONH blood flow and visual field loss in NTG, further linking systemic oxidative burden to IOP-independent glaucomatous progression [55]. Collectively, these findings indicate that glaucoma-associated systemic oxidative stress spans multiple biomarker classes and that its contribution to structural and functional damage varies by disease subtype, age, and sex.

### 6.3. Other Systemic and Structural Surrogate Markers

Beyond circulating redox markers, mitochondrial respiratory function in peripheral blood mononuclear cells (PBMCs) has emerged as a clinically relevant systemic biomarker. Petriti and colleagues demonstrated that PBMC oxygen consumption rate (OCR) is significantly lower in both HTG and NTG patients than in controls, with NTG showing the greater deficit, and that lower OCR is strongly associated with faster visual field progression in IOP-treated patients, explaining 13% of progression variance—comparable to the 16% explained by IOP itself in an untreated reference cohort [56]. This finding is mechanistically grounded in earlier work by Abu-Amero and colleagues, who showed that mitochondrial respiratory activity is reduced by 21% in the lymphocytes of POAG patients, accompanied by a spectrum of novel nonsynonymous mtDNA changes—predominantly transversions implying oxidative stress—found exclusively in POAG and not in controls [57].

Systemic oxidative stress also associates with structural surrogates of glaucomatous vulnerability. Uchida and colleagues found that serum BAP independently predicts corneal hysteresis in older female OAG patients, suggesting that oxidative burden may contribute to the biomechanical compromise of ocular connective tissues in a sex- and age-dependent manner [58]. At the level of retinal structure, Takahashi and colleagues identified BAP as an independent correlate of visual acuity decline in OAG patients with loss of macular GCC thickness at the papillomacular bundle, alongside corneal hysteresis and optic nerve head blood flow, highlighting the multifactorial interplay between systemic oxidative status, structural neurodegeneration, and functional vision loss [59]. Collectively, glaucoma-related oxidative burden extends across cellular bioenergetics, connective tissue biomechanics, and structural neurodegeneration.

**Table 1:** Oxidative stress biomarkers in glaucoma by sampling compartment. (Direction indicates change in glaucoma patients relative to controls. ↑ elevation; ↓ reduction).

Biomarkers	Direction	Compartment	Glaucoma Subtype	Key Finding/Reference
**Aqueous humor**				
MDA	↑	Aqueous humor	POAG	Elevated vs. cataract controls; also elevated in paired blood samples. (Nucci et al. *Mol Vis* 2013 [19])
TAC (TRAP)	↓	Aqueous humor	POAG	Reduced total antioxidant capacity; by 64% lower than controls by TRAP assay. (Ferreira et al. *Am J Ophthalmol* 2004 [10])
TAC	↓	Aqueous humor	POAG	Reduced by ORAC method; confirms antioxidant depletion across an independent cohort. (Nucci et al. *Mol Vis* 2013 [19])
8-OHdG	↑	Aqueous humor	POAG (severe stage)	Significantly higher in Black vs. White patients with severe POAG (*p* = 0.024); no significant difference in earlier stages. (Wu et al. *Ophthalmol Sci* 2022 [49])
**Plasma/Serum**				
MDA	↑	Serum	POAG	Elevated vs. non-glaucomatous cataract controls; also elevated in paired blood samples. (Rokicki et al. *BMC Ophthalmol* 2017 [21]; Nucci et al. *Mol Vis* 2013 [19])
GSH	↓	Blood (whole)	POAG and NTG	Blood GSH reduced similarly in both POAG and NTG vs. controls; GSH/GSSG redox index reduced only in POAG. (Gherghel et al. *IOVS* 2013 [22])
Total SOD	↓	Serum	POAG	Total SOD activity reduced (*p* = 0.003); Cu, Zn-SOD subtype not significantly different from controls. (Rokicki et al. *BMC Ophthalmol* 2017 [21])
Vitamin C	↓	Serum	NTG	Selectively reduced in NTG (*p* = 0.04); vitamins A, D, E not significantly different. (Yuki et al. *Graefes Arch* 2010 [50])
Uric acid	↑	Serum	NTG	Paradoxically elevated in NTG (*p* = 0.01); may reflect compensatory antioxidant upregulation. (Yuki et al. *Graefes Arch* 2010 [50])
Nicotinamide	↓	Plasma	POAG	~30% reduction vs. controls; replicated in independent cohort; implies impaired NAD^+^ salvage pathway. (Kouassi Nzoughet et al. *IOVS* 2019 [51])
BAP (biological antioxidant potential)	↓	Serum	POAG and XFG	BAP reduced in POAG and exfoliation glaucoma (*p* = 0.0062); dROM and SH not significantly different. (Tanito et al. *PLoS ONE* 2012 [52])
BAP	↓	Serum	OAG (young males, ≤65 yr)	Lower serum BAP associated with reduced estimated RGC counts, particularly in males aged ≤65. (Asano et al. *Sci Rep* 2017 [53])
BAP	↓	Serum	NTG (males)	Lower BAP associated with reduced optic nerve head blood flow in male NTG patients only; not significant in females. (Sato et al. *PLoS ONE* 2023 [54])
**Systemic and structural surrogate markers**				
Urinary 8-OHdG; skin autofluorescence (SAF)	↑	Urine; skin	NTG	Both correlated with reduced ONH tissue-area blood flow; SAF also associated with VF mean deviation and RNFL thickness. (Himori et al. *Graefes Arch* 2016 [55])
Mitochondrial respiratory activity (MRA)	↓	Peripheral lymphocytes	POAG	MRA decreased by 21% vs. controls (*p* < 0.001); multiple mtDNA transversion mutations identified, consistent with oxidative mtDNA damage. (Abu-Amero et al. *IOVS* 2006 [57])
PBMC oxygen consumption rate (OCR)	↓	PBMCs	HTG and NTG	OCR reduced in both HTG and NTG; lower OCR strongly associated with faster VF progression under IOP-lowering treatment (*p* < 0.001). (Petriti et al. *Nat Med* 2024 [56])
Corneal hysteresis (CH)	—	Anterior segment (biomechanical)	OAG (females >57 yr)	Lower serum BAP independently predicted reduced corneal hysteresis in women over 57 with OAG; not significant in younger patients or males. (Uchida et al. *Sci Rep* 2020 [58])
Macular GCC thickness	—	Retinal structure (OCT)	Glaucoma (including NTG)	GCC thinning (nasal sector at 9 o’clock) associated with visual acuity decline; lower BAP and corneal hysteresis were independent predictors of BCVA loss. (Takahashi et al. *TVST* 2023 [59])

BAP, biological antioxidant potential; CH, corneal hysteresis; GCC, ganglion cell complex; GSH, glutathione; GSSG, glutathione disulfide; IOP, intraocular pressure; MDA, malondialdehyde; MRA, Mitochondrial respiratory activity; NAD^+^, nicotinamide adenine dinucleotide; NTG, normal-tension glaucoma; OCR, oxygen consumption rate; ONH, optic nerve head; PBMC, peripheral blood mononuclear cells; POAG, primary open-angle glaucoma; RGC, retinal ganglion cell; RNFL, retinal nerve fiber layer; SAF, skin autofluorescence; SH, sulfhydryl groups; SOD, superoxide dismutase; TAC, total antioxidant capacity; VF, visual field; 8-OHdG, 8-hydroxy-2′-deoxyguanosine; XFG: exfoliation glaucoma.

### 6.4. Systemic Comorbidities and Their Management as Modifiers of the Oxidative Burden in POAG

POAG rarely occurs in isolation. Epidemiological data from both Asian and European populations show that most patients carry at least one cardiometabolic comorbidity, with hypertension present in roughly 50 to 75% of cases, dyslipidemia in 30 to 50%, and diabetes mellitus in about 30% [60,61]. This is relevant to the present review because all three conditions are themselves established sources of systemic oxidative stress. Hypertension increases vascular ROS production through angiotensin II signaling and NADPH oxidase activation [62], type 2 diabetes combines oxidative stress with endoplasmic reticulum stress and chronic inflammation [63], and dyslipidemia and atherosclerosis generate a comparable response through oxidized LDL and mitochondrial damage-associated molecular patterns [64].

An important qualification is that POAG is itself a strongly age-related disease, and the prevalence of all three comorbidities likewise increases with advancing age. Age is therefore a shared risk factor, and the systemic oxidative changes seen in glaucoma patients reflect, in part, the same age-dependent decline in redox homeostasis that drives these cardiometabolic conditions, as discussed in relation to the aging eye in Section 3.2. Their frequent co-occurrence in POAG is thus expected on the basis of shared aging rather than implying a glaucoma-specific mechanism.

This overlap affects how the systemic biomarker data in Section 6.2 and Section 6.3 should be interpreted. Because these comorbidities raise circulating oxidative markers on their own, part of the systemic oxidative signal reported in POAG cohorts may reflect coexisting cardiometabolic disease rather than glaucoma itself. Differences in comorbidity prevalence across study populations may also contribute to the inconsistent direction of serum antioxidant enzyme activity seen between reports. Biomarker studies in glaucoma should therefore record these comorbidities and adjust for them, along with the relevant medications, in their analyses.

The treatment of these conditions deserves separate consideration. Renin-angiotensin system inhibitors, statins, and several glucose-lowering agents have antioxidant and anti-inflammatory effects that go beyond their primary indication, so routine guideline-based management may lower systemic oxidative stress as well. Whether better control of cardiometabolic comorbidity also benefits the IOP-independent oxidative component of POAG is a reasonable question, but one that has not been tested against glaucoma-specific structural or functional endpoints and would be subject to confounding by indication. At this stage, comorbidity is best handled as a variable to control for in oxidative-stress research and as a plausible modifiable factor worth evaluating prospectively.

## 7. Antioxidant Therapeutic Strategies: Preclinical Evidence and Clinical Translation

The mechanistic evidence reviewed above has motivated a wide-ranging search for antioxidant-based neuroprotective strategies in glaucoma. Below, we review the most clinically advanced candidates, organized by mechanism, and critically evaluate the degree to which preclinical promise has been translated to human efficacy data (Table 2).

### 7.1. Nicotinamide (Vitamin B3) and NAD+ Replenishment

Nicotinamide (NAM, niacinamide) is the amide form of vitamin B3 and a direct precursor of NAD^+^ via the salvage pathway catalyzed by nicotinamide phosphoribosyltransferase (NAMPT). Its rationale in glaucoma is grounded in the finding that retinal NAD+ levels decline with age and correlate with IOP-related vulnerability of RGCs in both DBA/2J spontaneous glaucoma mice and inducible ocular hypertension models [65]. In DBA/2J mice, dietary supplementation with high-dose NAM was remarkable: the highest tested dose resulted in 93% of eyes failing to develop glaucomatous degeneration—a prevention rate that prompted rapid clinical translation [65].

In a human crossover randomized trial, oral NAM (1.5 g/day escalating to 3 g/day over 12 weeks) significantly improved inner retinal electrophysiological function as measured by the photopic negative response amplitude compared to placebo, without altering IOP, suggesting a direct metabolic–neuroprotective effect [66]. Several Phase II/III trials are now underway, including the Nicotinamide in Glaucoma Trial (NCT05405868) and a Singapore-based parallel RCT, examining whether NAM slows visual field mean deviation decline over 18–27 months in newly diagnosed glaucoma patients receiving 1.5–3 g/day [67].

The safety profile of NAM at clinical trial doses has generally been favorable; however, cases of drug-induced liver injury (DILI) have been reported in two participants across these trials—one in the United States (hepatotoxicity with normalization on discontinuation) and one in Singapore (DILI with autoimmune features)—prompting the American Glaucoma Society and American Academy of Ophthalmology (AGS/AAO) to issue a joint 2025 position statement recommending periodic liver function monitoring for patients using NAM at doses approaching 3 g/day [68]. This safety signal, while rare, underscores the importance of rigorous pharmacovigilance in repurposing metabolic interventions for chronic neurological conditions. Recent narrative syntheses emphasize that, despite encouraging electrophysiological and visual-field signals, adequately powered trials defining dose, duration, and clinical endpoints remain absent, with multiple long-term NAM and NAD-precursor trials now ongoing [69,70].

### 7.2. Coenzyme Q10 (CoQ10, Ubiquinol)

CoQ10 functions as a critical electron carrier in the mitochondrial ETC (transferring electrons from complexes I and II to complex III) and as a lipid-soluble antioxidant that scavenges lipid peroxyl radicals. Its concentration in ocular tissues declines with age, and reduced CoQ10 levels have been implicated in RGC vulnerability [71]. In rodent models of glaucoma, CoQ10 administration inhibited glutamate excitotoxicity, preserved mitochondrial membrane potential, and reduced oxidative stress-mediated RGC apoptosis [71]. A topical CoQ10/vitamin E formulation has been investigated in small clinical trials and showed improvements in pattern electroretinogram (PERG) amplitude, pattern visual evoked potentials (PVEP), and contrast sensitivity in early POAG, without IOP changes [72].

A combination neuroprotection approach utilizing CoQ10 with citicoline has been proposed, with additive electrophysiological benefits reported in preliminary clinical studies, reflecting complementary mechanisms: CoQ10 addressing the mitochondrial–redox dimension and citicoline the structural–membrane dimension of RGC vulnerability [72].

### 7.3. Nrf2 Activators: Sulforaphane and Triterpenoids

Pharmacological activation of the Nrf2/ARE pathway represents a mechanistically attractive strategy for broad-spectrum antioxidant neuroprotection, as it induces an endogenous, self-limiting and adaptive transcriptional program rather than delivering a single exogenous scavenger. Evidence that this pathway is altered in patients supports such a strategy, as Nrf2 is downregulated in glaucomatous relative to normal human trabecular meshwork [46]. Sulforaphane (SFN), an isothiocyanate derived from broccoli sprouts, is one of the most potent naturally occurring Nrf2 activators, covalently modifying Keap1 cysteine residues to release and activate Nrf2 [73]. In animal models, SFN has been shown to protect TM cells from H_2_O_2_-induced apoptosis [74]. The synthetic triterpenoid CDDO-Im demonstrated neuroprotective effects against optic nerve crush-induced RGC loss in WT mice, with Nrf2 knockout mice showing significantly greater RGC loss, further validating the pathway as a therapeutic target [42]. Human clinical trials of Nrf2-activating compounds specifically in glaucoma are limited, although trials for related neurodegenerative conditions (e.g., Alzheimer’s disease) may inform dose-finding and target engagement.

### 7.4. Alpha-Lipoic Acid (ALA)

ALA is a disulfide compound synthesized in the mitochondria that serves as a cofactor for pyruvate dehydrogenase and alpha-ketoglutarate dehydrogenase. As both a water- and lipid-soluble antioxidant, it is uniquely capable of distributing across cellular and mitochondrial membranes. ALA recycles ascorbate, vitamin E, and GSH from their oxidized forms, placing it at the intersection of the redox and metabolic neuroprotection strategies [75]. Although clinical data in glaucoma specifically are limited, ALA has shown neuroprotective effects in diabetic retinopathy models and may be particularly relevant in glaucoma subtypes with prominent metabolic comorbidity.

### 7.5. Mitochondria-Targeted Antioxidants: MitoQ

MitoQ (mitoquinone mesylate) is a coenzyme Q10 derivative covalently linked to a triphenylphosphonium (TPP+) cation, which drives its accumulation to several hundred-fold within the mitochondrial matrix—the primary site of ROS generation [76]. In a rat model of retinal ischemia–reperfusion injury induced by transient elevation of intraocular pressure to 110 mmHg, intravitreal MitoQ improved retinal function on dark-adapted flash electroretinography, increasing both a-wave and b-wave amplitudes in a dose-dependent manner, with the maximal effect at a final intravitreal concentration of 400 nM and daily dosing assessed on day 7 [76]. MitoQ also reduced retinal ROS generation, increased SOD1, and suppressed inflammatory and apoptotic signaling, acting through the SIRT1/Notch1/NADPH oxidase axis by upregulating SIRT1 while lowering cleaved-Notch1, Hes1, NOX1, and NOX4; these effects were abolished by the SIRT1 inhibitor EX527. While clinical trials in glaucoma have not yet been completed, the mitochondria-targeted delivery concept addresses a key pharmacokinetic limitation of conventional antioxidants—their inability to achieve therapeutic concentrations at the site of predominant ROS production within RGCs.

### 7.6. Resveratrol, Polyphenolic Compounds, and Dietary Antioxidant Supplementation

Resveratrol, a stilbene polyphenol found in red grapes and berries, activates SIRT1-dependent mitochondrial biogenesis and antioxidant pathways and has been shown to inhibit H_2_O_2_-induced apoptosis in RGC-5 cultures and TM cells. Curcumin, a polyphenolic curcuminoid, has demonstrated neuroprotective efficacy in topical nanocarrier formulations in rodent models of glaucoma, where twice-daily application of curcumin-loaded nanoparticles significantly reduced RGC loss in both ocular hypertension and partial optic nerve transection models without altering IOP [77]. The combination of multiple neuroprotective agents targeting complementary redox and apoptotic pathways—such as citicoline with CoQ10—has shown additive preclinical and clinical benefits, consistent with the view that multi-target strategies may outperform monotherapy.

Translational evidence supporting dietary antioxidant supplementation in glaucoma patients is beginning to emerge from Japanese clinical studies. Maekawa et al. demonstrated that a plant-derived oral antioxidant supplement—containing hesperidin, crocetin, and Tamarindus indica—prevented RGC loss in NMDA-injured mice by suppressing lipid peroxidation and excessive calpain activation [36]. Himori et al. subsequently conducted a prospective single-arm study in which the same three-component formulation was administered to 30 NTG patients for eight weeks, with follow-up for an additional eight weeks; in the subgroup with relatively high baseline oxidative stress, urinary 8-OHdG was significantly reduced and BAP was significantly elevated, although no significant changes in visual field parameters were reported [78]. While the trial was modest in scale, it represents one of the few prospective human data sets linking antioxidant supplementation directly to both biomarker and functional endpoints in glaucoma. Nutritional interventions targeting ocular vascular supply have also been explored: a ginger extract (6-shogaol-enriched fraction) administered to rats with endothelin-induced retinal blood flow dysfunction significantly improved optic nerve head perfusion [79], which was also examined in a population-based observational study [80], providing a mechanistic rationale for the investigation of vasoactive phytochemicals as complementary neuroprotective agents in vascular-predominant glaucoma phenotypes such as NTG. Clinical evidence for polyphenolic compounds in glaucoma, however, remains largely observational or based on small, short-duration trials.

### 7.7. Ginkgo Biloba Extract

Ginkgo biloba leaf extract (EGb 761) possesses antioxidant, anti-apoptotic, and vasodilatory properties, including inhibition of platelet-activating factor and free radical scavenging by its flavonoid and terpenoid constituents [81]. Two small NTG trials have reported clinical benefits, though with important methodological differences [81,82]. Quaranta et al. conducted a prospective, randomized, placebo-controlled, double-masked crossover trial in 27 NTG patients, demonstrating significant improvement in preexisting visual field indices after 40 mg EGb 761 three times daily for 4 weeks; the effect was most pronounced in patients with concomitant Raynaud’s syndrome, suggesting particular benefit in those with systemic vasospastic disease [81]. Lee et al. retrospectively evaluated 42 NTG eyes treated with 80 mg twice daily over a mean follow-up of 12.3 years, reporting that the regression coefficients for mean deviation, pattern standard deviation, and visual field index improved significantly after treatment initiation, indicating slowed progression rather than acute reversal of existing defects [82]. A contradicting randomized crossover trial in Chinese NTG patients found no benefit on mean defect or contrast sensitivity, underscoring the inconsistency in the current evidence [83]. No large prospective RCT has been conducted. Ginkgo biloba is generally well tolerated, but clinicians should note that, while controlled studies using EGb 761 have not consistently demonstrated clinically significant effects on hemostasis, caution is advisable when combining Ginkgo biloba with antiplatelet agents (particularly clopidogrel and aspirin), anticoagulants, or NSAIDs due to case reports and pharmacodynamic plausibility; an interaction with MAO-inhibiting drugs has been suggested in in vitro studies but lacks robust clinical confirmation; a theoretical interaction with monoamine-oxidase-inhibiting antidepressants has also been raised. A mild hypoglycemic effect warrants monitoring in diabetic patients. The combination of EGb 761 with other antioxidant neuroprotectants merits further investigation.

**Table 2:** Antioxidant-based neuroprotective strategies in glaucoma: mechanisms, evidence, and safety.

Agent	Primary Mechanism	Best Preclinical Evidence	Evidence Level	Key Clinical Study/Trial	Safety/Notes
Nicotinamide (NAM; vitamin B3)	NAD^+^ repletion via salvage pathway (NAMPT); mitochondrial ETC support; SIRT1 activation	93% of eyes free from glaucoma at highest dose, occurring despite elevated IOP [65].	Pilot RCT	Crossover RCT (n = 57): improved PhNR amplitude (inner retinal function by ERG) at 1.5 → 3 g/day dose escalation; trend for improved VF mean deviation [66].	DILI at ≥3 g/day (2 cases across trials); periodic LFT monitoring recommended [68].
Coenzyme Q10 (CoQ10)	Mitochondrial ETC electron carrier (complex I&II → III); lipid-soluble ROS scavenger	Inhibits glutamate excitotoxicity; preserves ΔΨm in rodent glaucoma model [71].	Non-randomized control trial	Topical CoQ10/vit E: improved PERG and VEP responses vs. β-blocker alone in OAG [84].	Generally well tolerated in short-term trials; no serious adverse events reported to date; larger trials ongoing.
Sulforaphane (SFN)	Keap1 Cys adduction → Nrf2 nuclear translocation → HO-1, NQO1, SOD2 transcription	Retinal I-R: Nrf2/HO-1 activation, ↓ RGC loss [85]; TM H_2_O_2_ protection in vitro [74].	Preclinical	No glaucoma-specific RCT; neurodegenerative disease trials inform dose-finding.	Generally safe at dietary doses. Bioavailability highly variable due to gut microbiota-dependent conversion of glucoraphanin to SFN (myrosinase activity).
Alpha-lipoic acid (ALA)	Mitochondrial cofactor; recycles ascorbate, vitamin E, and glutathione [86]	DBA/2J glaucoma mice (dietary ALA): significantly higher RGC density; ↓ lipid peroxidation, 3-NT, 8-OHdG, NOS-2 [87].	Preclinical	No completed glaucoma efficacy RCT.	Dual water/lipid solubility enables distribution across cellular membranes including to mitochondrial matrix.
MitoQ (mitoquinone)	TPP^+^-linked CoQ10; several-hundred-fold mitochondrial matrix accumulation; site-specific ROS scavenging at ETC [88]	Retinal I-R (rat): improved retinal function, ↓ ROS, modulation of SIRT1/Notch1/NOX pathway [76].	Phase II completed (PD [89] , hepatitis C [90] ); no ocular RCT	No completed glaucoma RCT; Phase II PD trial. Cardiovascular/metabolic disease trials are ongoing.	Addresses bioavailability limitation of conventional CoQ10; oral route feasible.
Resveratrol	SIRT1 activation → mitochondrial biogenesis; NLRP3 inflammasome suppression; Keap1/Nrf2/HO-1 activation; MAPK (p38/ERK/JNK) inhibition in RGCs.	Retinal I-R (mouse): dual anti-inflammatory + antioxidant effects [39]; RGC-5 apoptosis inhibition.	Preclinical	Preclinical synergy with CoQ10 or curcumin; not yet studied in glaucoma patients.	Low oral bioavailability; nanoformulation required for adequate intraocular delivery.
Curcumin	NF-κB inhibition; Nrf2 activation; anti-apoptotic (Bcl-2 upregulation); multi-target redox modulation	Topical nanocarriers: significant RGC neuroprotection in rodent glaucoma models [77].	Preclinical	No completed glaucoma RCT; nanocarrier formulation required for clinically relevant ocular bioavailability.	Poor aqueous solubility: nanoparticle formulation required; generally safe.
Multi-ingredient dietary antioxidant supplement	Combined Hesperidin (bioflavonoid) + crocetin (apocarotenoid) + *Tamarindus indica* extract; multiple antioxidant pathways simultaneously	Plant-derived supplement prevents RGC loss in NMDA-injured mice [36].	Pilot clinical	8-week supplementation associated with ↓ systemic oxidative stress markers in patients with high baseline OS; no VF outcome reported [78].	Good tolerability; limited by small sample size and absence of randomization.
Ginkgo biloba extract (EGb 761)	Flavonoid/terpenoid ROS scavenging; PAF inhibition; vasodilation → ↑ ONH blood flow	Rat chronic glaucoma model (GBE 761) protects RGCs from degeneration [91]; optic nerve crush model: GBE preserved RGC survival [92].	Pilot RCT, retrospective study	Small NTG trials: improved preexisting VF defects [81] and VF progression slowed [82]; contradicting RCT [83].	Well tolerated; drug interactions: bleeding risk increase with anticoagulants, hypoglycemic effect in diabetes.
Nicotinamide riboside (NR)	NAD^+^ precursor via NRK1/2 pathway; alternative to NAM with potentially superior tolerability profile	Improves mitochondrial function in animal models; well-tolerated in human pharmacokinetic studies [93].	Phase 2 RCT completed (negative result)	Completed RCT (INSIGHT; 300 mg/day vs. placebo, 24 months): no significant difference in RNFL thinning or visual field index; subgroup analysis showed faster RNFL/VFI decline in older NR-treated patients [94,95].	NAD^+^ doubling at steady state confirmed; long-term safety in glaucoma context unknown.

DILI, drug-induced liver injury; ETC, electron transport chain; HO-1, heme oxygenase-1; I-R, ischemia–reperfusion; LFT, liver function test; NAD^+^, nicotinamide adenine dinucleotide; NAMPT, nicotinamide phosphoribosyltransferase; NMDA, N-methyl-d-aspartate; NQO1, NADPH quinone oxidoreductase 1; NTG, normal-tension glaucoma; ONH, optic nerve head; PAF, platelet-activating factor; PERG, pattern electroretinogram; RGC, retinal ganglion cell; ROS, reactive oxygen species; SIRT1, sirtuin-1; SOD2, superoxide dismutase 2; TM, trabecular meshwork; TPP^+^, triphenylphosphonium; VEP, visual evoked potential; VF, visual field; ΔΨm, mitochondrial membrane potential; **↑, **elevation**; ↓, **reduction**.**

## 8. Drug Delivery Innovations for Ocular Antioxidant Therapy

A persistent challenge in translating antioxidant therapies to clinical practice is achieving adequate tissue concentrations at the relevant ocular targets—primarily the TM for anterior segment protection and the inner retina for RGC neuroprotection. Systemic administration of antioxidants is limited by the blood–retinal barrier, hepatic first-pass metabolism, and the low aqueous bioavailability of many natural compounds. Topical ophthalmic formulations face challenges of corneal penetration, lacrimation, and nasolacrimal drainage.

Nanoparticle-based drug delivery systems—including polymeric nanoparticles, liposomes, solid lipid nanoparticles, and dendrimers—have been extensively investigated as vehicles to overcome these barriers [96]. Topical curcumin nanocarriers demonstrated superior corneal penetration and significant neuroprotection in rodent glaucoma models compared with conventional curcumin formulations [77]. Similarly, biodegradable polymeric microspheres and nanoparticles co-loaded with CoQ10, resveratrol, and other neuroprotective agents have shown sustained intraocular release profiles and improved RGC survival in experimental models. Intravitreal sustained-release implants, which bypass the blood–retinal barrier entirely, are an additional modality currently under investigation for antioxidant compound delivery to the posterior segment.

Gene therapy approaches offer a complementary strategy by inducing endogenous antioxidant production directly within RGCs or glial cells. AAV-mediated delivery of the Nrf2 gene, NMNAT1 (which drives NAD^+^ synthesis), or antioxidant enzymes (SOD2, catalase) to the retina has demonstrated compelling RGC neuroprotection in rodent models, and Phase I/II human trials are underway for related antioxidant and neuroprotective gene therapy approaches in other ocular neurodegenerative diseases [97]. The integration of gene therapy with pharmacological antioxidant supplementation may ultimately prove most effective for patients with advanced disease or genetically defined high-risk profiles.

## 9. Interaction with IOP-Lowering Therapy

A conceptually important question is whether antioxidant therapies should be understood as adjuncts to IOP reduction or as parallel, independent treatment axes. The evidence suggests they serve complementary roles. IOP elevation mechanically stresses RGC axons at the lamina cribrosa and simultaneously induces ischemia–reperfusion-mediated ROS production. IOP reduction therefore reduces both the mechanical and oxidative burden on RGCs. However, as discussed above, oxidative stress continues to operate in the presence of well-controlled IOP, particularly in ageing patients with cumulative oxidative damage to the TM, and in NTG patients where vascular and metabolic mechanisms predominate [98].

Some IOP-lowering agents may themselves carry antioxidant properties. Brimonidine, an alpha-2 adrenergic agonist, was extensively studied as a neuroprotective agent partly based on evidence of its anti-apoptotic and antioxidant-like signaling in vitro. The Low-Pressure Glaucoma Treatment Study (LoGTS) found significantly less visual field progression in brimonidine-treated patients than in timolol-treated patients despite similar IOP lowering, raising the possibility of IOP-independent neuroprotection [99]. However, the high dropout rate in the brimonidine arm (55% did not complete the 4-year follow-up, predominantly due to ocular allergy), resulting in differential attrition, and consequent high risk of bias have led most systematic reviewers to conclude that the neuroprotective interpretation of these results remains unconfirmed. Latanoprost and other prostaglandin analogues may modulate neurotrophin signaling without direct ROS scavenging activity. The net implication is that antioxidant interventions are best positioned as additions to, rather than substitutes for, established IOP-lowering regimens.

## 10. Limitations, Gaps, and Future Directions

Despite the compelling mechanistic and preclinical data, several critical gaps impede the translation of antioxidant-based neuroprotection into clinical practice. First, most human studies measuring oxidative biomarkers in glaucoma are cross-sectional, precluding causal inference and limiting understanding of the temporal dynamics of oxidative stress across disease stages. Longitudinal cohort studies correlating serial biomarker measurements with structural (OCT retinal nerve fiber layer, optic disc morphology) and functional (visual field mean deviation) endpoints are needed. Second, the clinical trial evidence base for most antioxidant compounds in glaucoma remains inadequate. Trials have been predominantly small (fewer than 100 participants), short-duration (six to twelve months), and heterogeneous in participant selection, dosing regimens, and outcome measures. Rigorous, large-scale, multi-center randomized controlled trials with pre-specified primary endpoints—ideally visual field mean deviation change at 24 months or beyond—are essential to determine whether bioelectrophysiological improvements observed in early trials translate into clinically meaningful vision preservation. Third, there is a need for stage-specific therapeutic frameworks. The relative contribution of TM oxidative dysfunction versus RGC mitochondrial vulnerability likely varies across disease stages: early disease may benefit most from anterior segment-targeted interventions (TM antioxidants), while advanced disease requires direct RGC neuroprotection. Future trial designs should incorporate pre-stratification by structural and functional disease severity. Fourth, emerging data on racial disparities in oxidative burden [49] and the potential differential responsiveness to NAD+ supplementation across ancestries highlight the necessity for diverse, globally representative trial populations. Current trials have been predominantly conducted in populations of European descent. Fifth, the combination approach—simultaneously targeting multiple pathogenic nodes—warrants dedicated evaluation. Combinations such as NAM with CoQ10, or citicoline with Nrf2 activators, may have additive or synergistic effects in phase II proof-of-concept studies and are mechanistically well-justified. The development of validated composite neuroprotection endpoints that capture electrophysiological, structural, and patient-reported outcomes will be necessary to detect the modest incremental effects likely achievable with adjunct interventions. Finally, the DILI signal from nicotinamide trials [68] illustrates that even familiar vitamins carry hepatotoxic risk at the supratherapeutic doses required for ophthalmic neuroprotection, and mandates systematic pharmacovigilance infrastructure in all future trials. Optimal long-term dosing strategies, and the question of whether lower doses may be sufficient for maintenance neuroprotection once an initial high-dose induction phase has been completed, remain to be established.

## 11. Conclusions

Oxidative stress is not a peripheral or coincidental feature of glaucoma, but a central, mechanistically integrated driver of both anterior segment (TM dysfunction, elevated IOP) and posterior segment (RGC metabolic vulnerability, neurodegeneration) pathology. The evidence reviewed here—spanning aqueous humor biochemistry, animal models with defined genetic dissection of antioxidant pathways, systemic biomarker profiling, and early clinical trials—establishes a compelling case for oxidative stress as a viable therapeutic target in glaucoma, particularly for patients who continue to lose vision despite optimal IOP control.

The Nrf2/Keap1/ARE pathway and NAD^+^ metabolism represent the two most clinically advanced mechanistic frameworks for antioxidant intervention, with nicotinamide the most robustly supported agent by current preclinical and early clinical data. Coenzyme Q10, alpha-lipoic acid, mitochondria-targeted antioxidants such as MitoQ, and Nrf2-activating phytochemicals represent an expanding therapeutic toolkit whose clinical profiles are still being defined. Innovations in drug delivery—nanoparticle carriers, sustained-release implants, and AAV-based gene therapy—will be critical enablers of effective posterior segment antioxidant delivery.

The field now stands at a critical inflection point: the mechanistic foundations are sufficiently robust to justify, and the unmet clinical need sufficiently pressing to demand, adequately powered, long-term, randomized clinical trials. Advancing oxidative stress modulation from a promising adjunct to an established standard of care in glaucoma management will require not only rigorous trial design but also a precision medicine framework that identifies which patients—by disease stage, genetic profile, biomarker signature, and ancestry—are most likely to benefit.

## Data Availability

No new data were created or analyzed in this study. Data sharing is not applicable to this article.

## Abbreviations

The following abbreviations are used in this manuscript:

8-OHdG	8-hydroxy-2′-deoxyguanosine
4-HNE	4-hydroxynonenal
AAV	Adeno-associated virus
ANT	Adenine nucleotide translocase
AOPPs	Advanced oxidation protein products
ALA	Alpha-Lipoic Acid
AGS/AAO	American Glaucoma Society and American Academy of Ophthalmology
ARE	Antioxidant response element
BAP	Biological antioxidant potential
CH	Corneal hysteresis
CMV	Cytomegalovirus
DHA	Docosahexaenoic acid
DILI	Drug-induced liver injury
d-ROM	Derivatives of reactive oxygen metabolites
ETC	Electron transport chain
ECM	Extracellular matrix
GSH	Glutathione
GSSG	Glutathione disulfide
GPx	Glutathione peroxidase
IOP	Intraocular pressure
MDA	Malondialdehyde
MRA	Mitochondrial respiratory activity
mtDNA	Mitochondrial DNA
mPTP	Mitochondrial permeability transition pore
MitoQ	Mitoquinone mesylate
NAD	Nicotinamide adenine dinucleotide
NAM	Nicotinamide
NMDA	N-methyl-D-aspartate
NO	Nitric oxide
NOX	NADPH oxidase
NTG	Normal-tension glaucoma
OCR	Oxygen consumption rate
ONH	Optic nerve head
PBMC	Peripheral blood mononuclear cells
PERG	Pattern electroretinogram
POAG	Primary open-angle glaucoma
RGC	Retinal ganglion cell
ROS	Reactive oxygen species
SAF	Skin autofluorescence
SASP	Senescence-associated secretory phenotype
SFN	Sulforaphane
SH	Sulfhydryl groups
SOD	Superoxide dismutase
TMCs	Trabecular meshwork cells
TAC	Total antioxidant capacity
TRAP	Total reactive antioxidant potential
TM	Trabecular meshwork
UPR	Unfolded protein response
VF	Visual field
XFG	Exfoliation glaucoma
